# Difference between Day and Night Temperature (DIF) and Light Intensity Affect Growth and Photosynthetic Characteristics of *Panax ginseng* Meyer Sprouts

**DOI:** 10.3390/plants12152820

**Published:** 2023-07-30

**Authors:** Jinnan Song, Jingli Yang, Byoung Ryong Jeong

**Affiliations:** 1 Shandong Facility Horticulture Bioengineering Research Center, Jia Sixie College of Agriculture, Weifang University of Science and Technology, Shouguang 262700, China; jinnansong93@gmail.com (J.S.); yangmiaomiaode@gmail.com (J.Y.); 2 Department of Horticulture, Division of Applied Life Science (BK21 Four Program), Graduate School of Gyeongsang National University, Jinju 52828, Republic of Korea; 3 Institute of Agriculture and Life Science, Gyeongsang National University, Jinju 52828, Republic of Korea; 4 Research Institute of Life Science, Gyeongsang National University, Jinju 52828, Republic of Korea

**Keywords:** root growth rate, leaf development, shoot length, photosynthesis, greenness index

## Abstract

*Panax ginseng* sprouts (PGS) have the advantage of requiring short-term cultivation while maintaining higher ginsenoside contents than traditional ginseng seedlings. It is feasible to improve their yield capacity by manipulating physical factors such as temperature and light. This study therefore investigated the effects of the DIF (difference between day and night temperature) and LI (light intensity) on the growth and photosynthetic characteristics of PGS. To this end, four DIF treatments (18/22 °C, 20/20 °C, 22/22 °C, 22/18 °C), corresponding to two LI regimes (20 PPFD, 200 PPFD), were applied on one-year-old ginseng rootlets in closed-type plant production systems (CPPSs). The PGS had distinctly different responses to the eight treatments. In particular, we found that negative DIF considerably hampered the growth and development of roots, shoots, leaves, and photosynthesis, regardless of the LI considered. The PGS treated with 20/20 °C combined with 20 PPFD displayed the best root growth, shoot development, leaf area, as well as optimal photosynthetic ability. On the other hand, we further showed that the root growth rate was positively correlated with the stem diameter, leaf traits, and photosynthetic ability, whereas it was negatively correlated with the petiole length, stem length, and shoot length. Collectively, 20/20 °C combined with 20 PPFD was the optimal condition in the current study, and may be regarded as a successful strategy for large-scale productions of PGS.

## 1. Introduction

Ginseng (Panax ginseng C.A. Meyer) is an important slow-growing perennial herb that is cultivated for its potent, valuable roots in East Asia, particularly in China, Korea, and Japan [1]. Given the fact that ginseng roots contain various bioactive agents, such as ginsenosides, they have been used as a tonic or stimulant to boost vitality and ameliorate the immune system in humans [2,3]. However, a long reproduction process and obstacles associated with the propagation method present difficulties in ginseng production. Specifically, the cultivation of ginseng plants usually requires an average of 3 to 4 years for the roots to attain marketable sizes [4].

Recently, *Panax ginseng* sprouts (PGS) have come to be considered as a new medicinal vegetable, attracting customers’ interest because they have a short cultivation period while still containing high levels of beneficial compounds [5,6]. Intuitively, approximately one month of cultivation time could be required for the harvest of PGS, imparting high sustainability and competitiveness of marketed ginseng foods. Additionally, certain previously published studies have indicated that the PGS leaves had a higher concentration of ginsenoside than the roots [7,8]. Increasing numbers of customers are showing a preference for whole ginseng plants (or roots), rather than the extracts [9]. In addition, according to the Ginseng Industry Act, the prices of PGS are more than double than those of ginseng seedlings [10]. In this regard, the ginseng leaves are equally as important as the roots for the food industry. Therefore, the cultivation and production of high-quality PGS, characterized by bigger leaves and larger roots, can help improve the economic profits of ginseng.

Temperature exerts a substantial influence on the growth, quality, and productivity of plants. In particular, the difference between the day and night temperature (DIF) can significantly regulate a variety of aspects regarding the plant physiology and morphology. Intensive studies, covering a wide range of plants from vegetables to herbs, have shown that low day/high night temperature (negative DIF) could inhibit stem elongation and consequently suppress internode elongation, unlike zero DIF (constant temperature) and positive DIF (high day/low night temperature) [11,12,13]. Although a great number of reports have shown that negative or zero DIF counteract plant development, many producers have adopted this method to maintain the stem elongation, in particular of herb production [13,14]. More compact plants of Begonia were obtained after, treatment with negative DIF, as described by Jacobsen [15]. Jensen reported that zero DIF was a better treatment for shaping Kalanchoe with more compact morphology [16]. It has also been observed that lemon balm cultivated with negative and zero DIF displayed longer shoot lengths than those treated with positive DIF [13]. However, to date, such influences associated with the application of DIF on the growth and physiology of PGS have rarely been reported. Moreover, the optimal temperature pertaining to the physical properties and chemical reactions in ginseng is low, and large numbers of pioneer studies have been performed attempting to figure out the best conditions for rendering the highest ginsenoside production in ginseng hairy roots, neglecting the effects of DIF on PGS, as well as the subsequent improvements on PGS quality [17,18,19,20].

Apart from temperature, light is a well-known factor that not only affects ginseng output and quality, but is also directly correlated with photosynthetic activity [6,19,21,22]. Light intensity is of great importance for plant growth and the production of health-promoting phytochemicals, because ginseng is a semi-shade species [23]. Too much light illumination on ginseng plants can cause photoinhibition, photobleaching, and chlorosis, followed by disturbed photosynthesis and even leaf death [23,24,25]. Specifically, a threshold of less than 25% light intensity (about 500 μmol·m^−2^·s^−1^ PPFD-Photosynthetic Photon Flux Density) of full sunlight is safe for ginseng growth [23]. However, the optimal light intensity for ginseng plants varies according to the cultivation conditions, ginseng cultivar, and ginseng growth period. For instance, Jung adopted a light intensity of 200 PPFD as the recovery condition after high-light-intensity stress for controlled-environment-cultured ginseng roots [26], while a very low light intensity (20 PPFD) has been widely applied for large-scale output of commercial PGS, according to the Rural Development Administration (RDA) [27].

Nonetheless, data concerning the synergistic effects of DIF and light intensity on root growth, leaf morphological development, and photosynthetic capacity in PGS remain incipient, especially in controlled environments. Recently, more and more attention has been focused on PGS production in plant factories with artificial lighting (PFAL), specifically on the delivery of uniform and pesticide-free transplants [6,22,24,28]. Therefore, closed-type plant production systems (CPPSs) make it possible to investigate the optimal DIF and light intensity for the production of high-quality PGS.

Therefore, the objective of this study on PGS is to (1) assess the combined effects of DIF and light intensity on physiology and morphology, (2) to accordingly determine the optimal treatment conditions that yield the most effective growth and photosynthetic ability, and to (3) concomitantly explore the associations among all the treatments and the parameters investigated.

## 2. Materials and Methods

### 2.1. Plant Materials and Culture Conditions

One-year-old ginseng roots (*Panax ginseng* C.A. Meyer) featuring similar morphologies consisting of a main tap root and a tiny emerging shoot were ordered from a ginseng grower in Geumsan, Chungnam, Korea and kept at 4 °C until use. The mean fresh weight and mean maximum root diameter were 0.68 g and 3.10 mm, respectively. The roots were planted in plant culture baskets (L × W × H, 53.3 × 27.3 × 6.3) filled with BVB medium (Bas Van Buuren Substrate, EN-12580, De Lier, The Netherlands) and moistened with running tap water. Then, the ginseng plants were cultivated in a controlled alternating diurnal environment consisting of 12 h of light (white LED) and 12 h of darkness at a constant temperature of 15 °C and relative humidity of 70% following Kim’s study [6], until the sprouts appeared. During this period, only tap water was applied to all the plants. Three days later, the germinated healthy ginseng roots with uniform morphology and without mechanical flaws were screened, selected, and transplanted to new plant culture baskets.

### 2.2. Treatments and Experimental Design

Subsequently, the transferred ginseng seedlings were divided equally into 8 parts and subjected to one of 8 treatments: Four day/night (18 °C/22 °C: −4DIF, 20 °C/20 °C: 0DIF, 22 °C/22 °C: 0DIF, 22 °C/18 °C: 4DIF) temperature regimes in combination with two light intensities (20 PPFD and 200 PPFD) for a 12 h/12 h day/night cycle. Thus, this experiment was laid out in a 2 × 4 factorial design. For each treatment, a completely randomized design with three biological replicates was adopted. For each replicate, 10 ginseng seedlings were used. A multipurpose nutrient solution (MNS) following our lab’s previous report [29] was applied as the only nutritional supply for the ginseng plants, 300 mL every three days. Four weeks later, the PGS with distinct phenotypes and responses to the treatments were individually observed and harvested.

### 2.3. Measurement of the Growth Parameters

The growth parameters were determined individually during harvest. The weights (root fresh weight and dry weight, shoot fresh weight and dry weight) were measured with an electronic balance. Root diameters and shoot diameters were recorded with a Vernier caliper (CD-20CPX, Mitutoyo Korea Co., Gunpo, Korea). The shoot length, stem length, leaf length and width, and petiole length were measured using a metal ruler. The leaf area data was collected with a leaf area scanning apparatus (LI-3000, Lincoln, NE, USA).

### 2.4. Calculations of the Relative Root Growth Rate

The relative root growth rate, consisting of the fresh weight and diameter, was calculated after the plants were harvested. The fresh weight and diameter of the ginseng roots were recorded individually before planting. After all the fresh weight and diameter data had been obtained, the relative root growth rate in fresh weight and diameter were calculated using the following formula:

Relative root growth rate (%) = (harvest value − original value)/original value × 100%

### 2.5. Determination of the Photosynthetic Characteristics

The leaf color values were evaluated using a color reader (CR-11, 1994 Minolta Co., Ltd., Osaka, Japan) and the corresponding color chart was obtained according to the ‘Munsell Color Palette System’ [30]. The SPAD (Soil and Plant Analysis Development) leaf greenness index was measured using a handheld device PolyPen RP 410 (Photon Systems Instruments, Drásov, Czech Republic). The chlorophyll contents were assayed based on Arnon’s study, with minor modifications [31]. In brief, 0.1 g of fresh plant leaves was submerged in 2 mL of the mixture medium (45% *v*/*v* ethanol, 45% *v*/*v* acetone, 10% *v*/*v* H_2_O) and incubated at 4 °C overnight. During the incubation, mild shaking was performed with a rotator (AG, FINEPCR, Seoul, Korea). Afterwards, the supernatant was transferred to the cuvette and the absorbance was read at 645 nm, 663 nm, and 440 nm using a spectrophotometer (Libra S22, Biochrom, Cambridge, UK). The chlorophyll—chlorophyll a, chlorophyll b, and carotenoids—contents were quantified individually using the following formulae:

$$\mathrm{Chlorophyll}\;a=\frac{\left(12.72\;\times \;\mathrm{OD}\;663\;-\;2.59\;\times \;\mathrm{OD}\;645\right)\;\times \;V}{\mathrm{Sample}\;\mathrm{fresh}\;\mathrm{weight}}\;\mathrm{Chlorophyll}\;b=\frac{\left(22.88\;\times \;\mathrm{OD}\;645\;-\;4.67\;\times \;\mathrm{OD}\;663\right)\;\times \;V}{\mathrm{Sample}\;\mathrm{fresh}\;\mathrm{weight}}\;\mathrm{Carotenoids}=\frac{4.7\;\times \;\mathrm{OD}\;440\;-\;0.27\;\times \;(\mathrm{Chl}\;a+\mathrm{Chl}\;b)}{\mathrm{Sample}\;\mathrm{fresh}\;\mathrm{weight}}$$

where ‘V’ is the volume of the extraction mixture solution used, and the chlorophyll content is expressed as milligram per gram of fresh leaf weight.

### 2.6. Statistical Analysis and Graphing

All data in this study are the means ± SE of more than three biological replications (n > 3). The data were analyzed using Duncan’s multiple comparison range test at *p* = 0.05 with the SAS statistical software 8.2 or unpaired two-tailed Mann–Whitney nonparametric test using GraphPad Prism 8.0.2 for the significant differences. The interaction between the DIF and light intensity (LI) treatments on the specific investigated traits were determined using two-way ANOVA ‘Linear models’ of *F*-test according to Fisher’s least significant difference (LSD) test (ns, *p* > 0.05; *, 0.01 < *p* < 0.05; **, *p* < 0.01; and ***, *p* < 0.001) with the SAS statistical software 8.2. Bar graphs were plotted using GraphPad Prism 8.2 software. The correlation heat map was generated and PCA (principal component analysis) was performed using the Origin 2022 program.

## 3. Results

### 3.1. Ginseng Growth in Terms of Morphology

**Figure 1:**
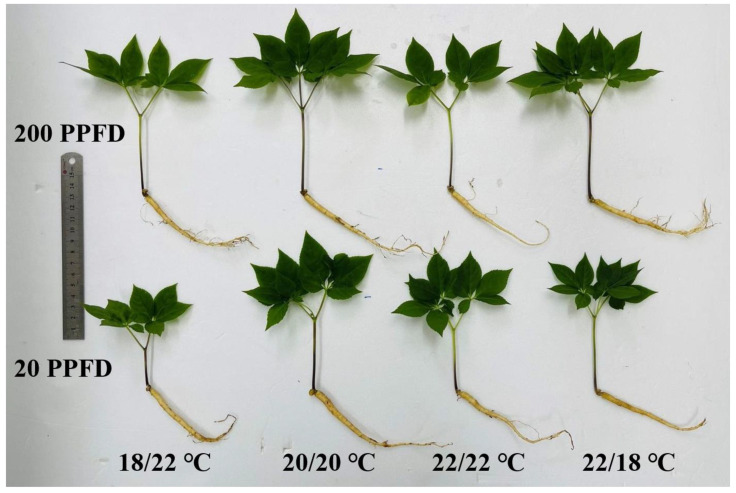
The effects of the eight treatments on the growth and morphology of ginseng sprouts.

The ginseng displayed distinct responses to the DIF and light intensity treatments (Figure 1). On the whole, regardless of the DIF, the shoot length and the petiole length were more prone to being greater when cultivated with high light intensity (200 PPFD) than when cultivated with low light intensity (20 PPFD). With the high light intensity, the roots were longer, but smaller in diameter. In addition, for the plants grown in the zero-DIF regimes (20/20 °C, 22/22 °C), the plants in 20 PPFD had notably larger leaves than those in 200 PPFD.

Similarly, regardless of the light intensity considered, negative DIF (18/22 °C) delivered stunted growth and disturbed morphology in the ginseng plants, as characterized by small roots and chlorosis in leaves. In contrast, the ginseng plants grown with zero DIF at 20/20 °C demonstrated better growth in the roots and shoots. Interestingly, the ginseng plants benefited dramatically more from high light intensity (200 PPFD) when treated in the 18/22 °C and 22/18 °C regimes. Overall, the collegial application of 20/20 °C and 20 PPFD resulted in the most prominent growth in ginseng.

### 3.2. Root Growth Traits

**Figure 2:**
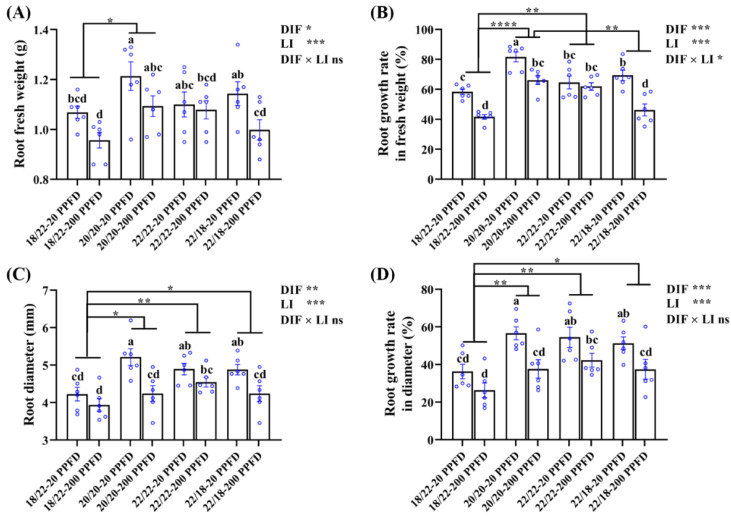
The effects of the eight treatments on the root growth traits regarding (**A**) root fresh weight, (**B**) root growth rate in fresh weight, (**C**) root diameter, and (**D**) root growth rate in diameter. The data displayed are the means ± SE of *n* = 6 replications by the blue circles. Different lowercase letters over bars indicate statistical differences (Duncan’s multiple comparison range test at *p* = 0.05); statistical differences between different DIF treatment groups are denoted by different numbers of asterisks (*, *p* < 0.05; **, *p* < 0.01; and ****, *p* < 0.0001 following unpaired two-tailed Mann–Whitney nonparametric test).

Consistent with the morphology depicted in Figure 1, the ginseng plants grown in the 20/20 °C in combination with a low light intensity at 20 PPFD exhibited significantly increased root fresh weight (10.2 and 6.1%, respectively) compared with that in plants grown in 22/22 °C-20 PPFD and 22/18 °C-20 PPFD (Figure 2A). Similarly, the root growth rate in terms of the fresh weight of the 20/20 °C-20 PPFD plants significantly improved (26.4 and 17.7%, respectively) compared to that in the 22/22 °C-20 PPFD and 22/18 °C-200 PPFD plants (Figure 2B). In addition, the root diameter of the plants grown in the 20/20 °C-20 PPFD markedly improved (by 6.7 and 7.2%, respectively) compared that in the 22/22 °C-20 PPFD and 22/18 °C-20 PPFD plants (Figure 2C). The root growth rate in terms of diameter of the 20/20 °C-20 PPFD plants significantly improved (4.0 and 10.5%, respectively) compared to that in the 22/22 °C-20 PPFD and 22/18 °C-200 PPFD plants (Figure 2D).

Furthermore, conspicuously, we found that both the DIF and light intensity (LI) remarkably affected root growth, while a significant interaction between them was found to affect root growth rate in fresh weight and root dry weight, rather than the other three parameters described herein (Figure 2B; Table 1).

### 3.3. Shoot Growth Traits

**Figure 3:**
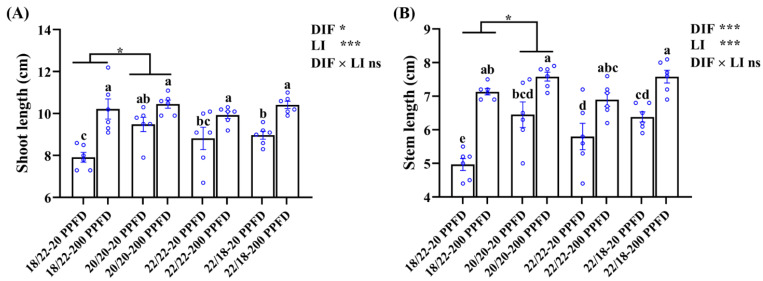
The effects of the eight treatments on the shoot growth characteristics regarding (**A**) shoot length, and (**B**) stem length. The exhibited data are the means ± SE of *n* = 6 replicates by the blue circles. Different lowercase letters accompanied by bars indicate statistical differences (Duncan’s multiple comparison range test at *p* = 0.05); statistical differences between the different DIF treatment groups are denoted by different numbers of stars (*, *p* < 0.05, unpaired two-tailed Mann–Whitney nonparametric test).

The ginseng shoot traits pertaining to the shoot length, stem length, shoot fresh weight and dry weight, and stem diameter are shown in Figure 3 and Table 1. The ginseng plants cultivated with a high light intensity of 200 PPFD displayed longer shoots and stems, regardless of the DIF treatment, and smaller values of shoot fresh weight, shoot dry weight, and stem diameter. For instance, in the 20/20 °C regime, the shoot length of plants grown with 200 PPFD were significantly (10.2%) greater than that in plants grown with 20 PPFD (Figure 3A). Similarly, the stem length in plants grown with 20/20 °C-200 PPFD was significantly increased by 17.5% compared to that in plants grown with 20/20 °C-20 PPFD (Figure 3B). Neither the DIF nor LI notably affected the fresh shoot weight (Table 1). There was a significant interaction between the DIF and LI affecting the shoot dry weight, while no influences were detected from this interaction on the other investigated shoot-related parameters (Figure 3; Table 1).

### 3.4. Leaf Growth Traits

**Figure 4:**
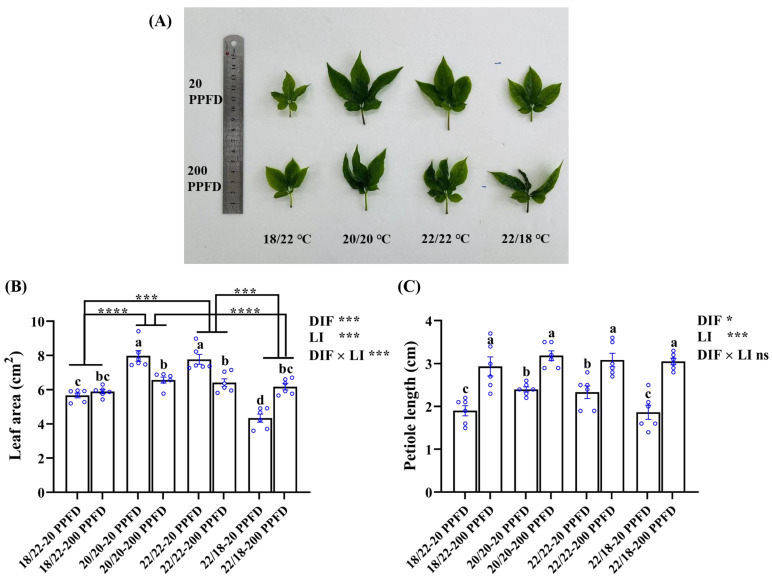
The responses of ginseng leaves, namely, (**A**) leaf morphology, (**B**) leaf area, and (**C**) petiole length, to the eight treatments. Data in (**B**) and (**C**) are the means ± SE of *n* = 6 replicates by the blue circles. Statistical differences of the eight treatments were determined by Duncan’s multiple range test at *p* = 0.05 and denoted by different lowercase letters over bars; statistical differences between different DIF treatment groups in (**B**) are differentiated by different numbers of asterisks (***, *p* < 0.001; ****, *p* < 0.0001; unpaired two-tailed Mann–Whitney nonparametric test).

Leaf-related characteristics, namely the leaf area, petiole length, and leaf length and width, were investigated further in response to the DIF and LI treatments (Figure 4; Table 1). Compared to the ginseng plants grown with negative and positive DIFs, those grown with zero DIF exhibited greater leaf areas when treated with 20 PPFD rather than with 200 PPFD (Figure 4A,B). It was observed that the leaf area in plants treated with 20/20 °C-20 PPFD and 22/22 °C-20 PPFD significantly improved by 21.5 and 21.1%, respectively, relative to that in plants treated with 20/20 °C-200 PPFD and 22/22 °C-200 PPFD (Figure 4B). Irrespective of the light intensity treatments, the leaf area of ginseng plants grown in zero-DIF regimes was significantly larger (Figure 4A,B). Taking the comparison between the 20/20 °C -20 PPFD and 22/18 °C -20 PPFD as an example, the leaf area of the former group was dramatically (83.9%) greater than that in the latter group (Figure 4B). However, regardless of the DIF treatment, the ginseng plants cultivated with a high light intensity (200 PPFD) showed longer petioles than those treated with a low light intensity (20 PPFD). Outstandingly, the petiole length in plants treated using the 22/18 °C-200 PPFD regime showed an improvement (64.0%) compared to that in plants treated using the 22/18 °C-20 PPFD regime (Figure 4C). In addition, significant interactions were detected between the DIF and LI in terms of the effect on the leaf length, width, and area, while little interactions conferred an effect on the petiole length (Figure 4; Table 1).

### 3.5. Leaf Color

To distinguish the leaf color differences in response to the eight treatments, we assessed and compared the leaf color against the ‘Munsell Color Palette System’ and further on the basis of leaf greenness index.

**Figure 5:**
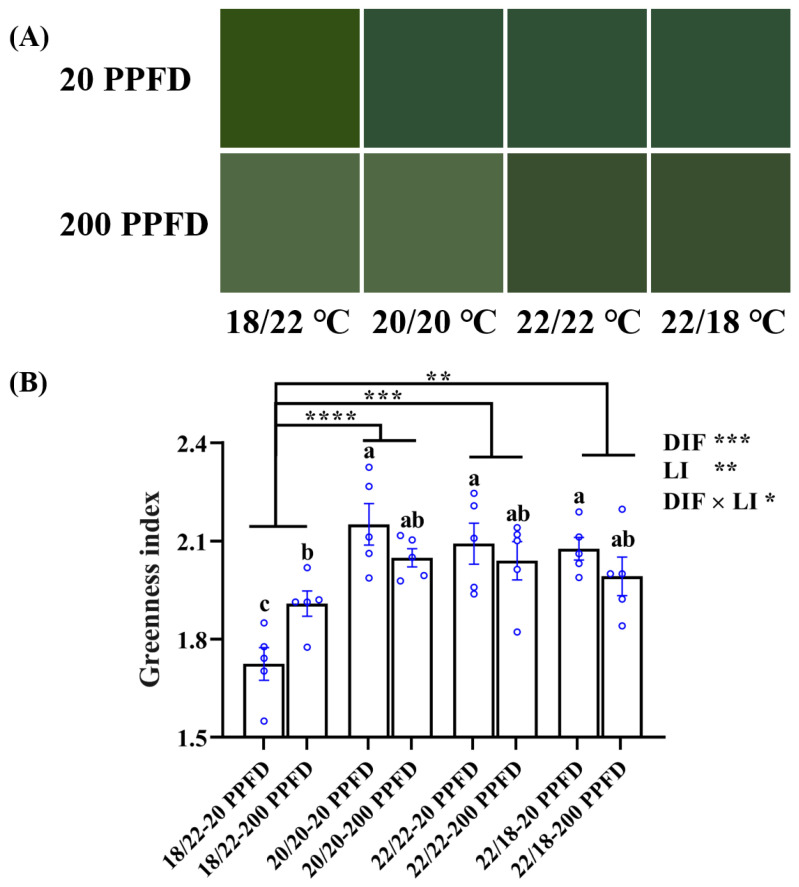
(**A**) Comparisons of the leaf color of ginseng plants in response to the 8 treatments and displayed in terms of (**B**) the greenness index. *n* = 5 replicates by the blue circles were averaged and statistically analyzed according to Duncan’s multiple comparison range test at *p* = 0.05 and indicated by different lowercase letters. Statistical differences between the different DIF treatments were determined with unpaired two-tailed Mann–Whitney nonparametric test, and indicated as *p* < 0.05 (*), < 0.01 (**), < 0.001 (***), and < 0.0001 (****). Error bars indicate the means ± SE.

As is apparent in Figure 5A, except for ginseng plants grown with a negative DIF (−4), 20 PPFD-treated plants were more prone to a light green color than those treated with 200 PPFD. Consistently, 20 PPFD-cultured plants in the 20/20 °C, 22/22 °C, and 22/18 °C displayed a significantly higher greenness index (5.5, 3.0, and 4.5%, respectively) than those in the 200 PPFD (Figure 5B). Moreover, irrespective of the light intensity treatment, both zero-DIF- and positive-DIF-cultured plants exhibited markedly greater greenness indices than those cultured in negative (−4) DIF. Concomitantly, a significant interaction between the DIF and LI on the greenness index was observed (Figure 5B).

### 3.6. Chlorophyll Contents

**Figure 6:**
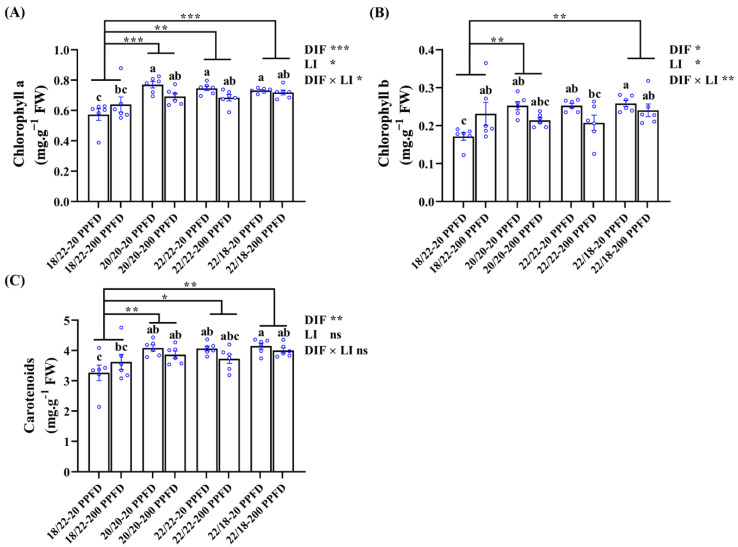
The effects of the DIF and LI treatments on the chlorophyll contents: (**A**) chlorophyll a, (**B**) chlorophyll b, and (**C**) carotenoids. Data displayed are the means ± SE of *n* = 6 replications by the blue circles and statistically analyzed following Duncan’s multiple comparison range test at *p* = 0.05 and denoted by different lowercase letters. Statistical differences between different DIF treatments are shown as *p* < 0.05 (*), <0.01 (**), and <0.001 (***) (unpaired two-tailed Mann–Whitney nonparametric analysis).

The chlorophyll a, b, and carotenoid contents in ginseng leaves subjected to the eight treatments were determined (Figure 6). Regardless of the light intensity treatment, chlorophyll contents in zero-DIF (20/20 °C, 22/22 °C) and positive-DIF (22/18 °C) plants were dramatically higher than those in negative-DIF plants (18/22). Meanwhile, ginseng plants treated with zero DIF and four DIF together with a low light intensity (20 PPFD) contained higher levels of chlorophyll. Specifically, for example, the plants grown in the 20/20 °C-20 PPFD and 20/20 °C-200 PPFD had drastically (35.1 and 21.1%, respectively) increased chlorophyll a content as compared to that in the 18/22 °C-20 PPFD; low-light-intensity-treated plants in the zero-DIF and positive-DIF regimes (20/20 °C-20 PPFD, 22/22 °C-20 PPFD, and 22/18 °C-20 PPFD) showed a distinct increase (11.6, 10.3, and 2.81%, respectively) in chlorophyll a content compared to those treated with high light intensities (20/20 °C-200 PPFD, 22/22 °C-200 PPFD, and 22/18 °C-200 PPFD).

In addition, DIF treatments conducted on ginseng plants displayed significant influences on all the chlorophyll levels, whereas light intensity failed to affect the carotenoid levels. Similarly, no interactions between the DIF and LI were observed to affect the carotenoid levels (Figure 6C).

### 3.7. Multivariate Analysis (Correlation Analysis and PCA)

To figure out the relationships among the root growth traits, leaf traits, and photosynthetic ability concurrently in order to visualize the responses of the studied parameters to the DIF and LI treatments, a multivariate analysis comprising a correlation analysis and principal component analysis (PCA) were carried out on the investigated parameters for the eight treatments.

**Figure 7:**
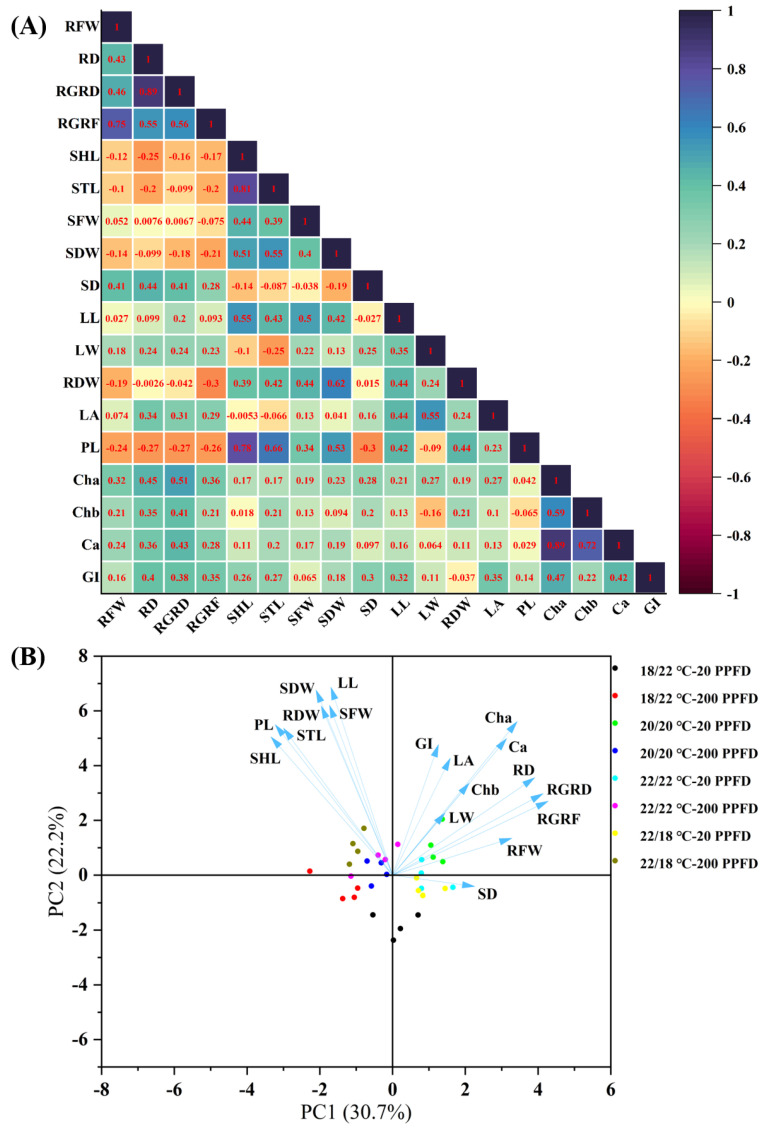
Multivariate data analysis showing (**A**) heatmap of correlation analysis and (**B**) PCA (principal component analysis) on the basis of the studied parameters regarding root growth, shoot growth, and photosynthetic traits subjected to the 8 treatments. RFW: root fresh weight; RD: root diameter; RGRD: root growth rate in diameter; RGRF: root growth rate in fresh weight; SHL: shoot length; STL: stem length; SFW: shoot fresh weight; SDW: shoot dry weight; SD: stem diameter; LL: leaf length; LW: leaf width; RDW: root dry weight; LA: leaf area; PL: petiole length; Cha: chlorophyll a; Chb: chlorophyll b; Ca: carotenoids; GI: greenness index.

According to the results of the multiple regression analysis, shown in Figure 7A, the root growth rate (in terms of fresh weight and diameter) is positively correlated with the stem diameter, leaf traits (length, width, and area), and photosynthetic ability (levels of chlorophyll a, b, carotenoids, and greenness index), whereas it is negatively correlated with the petiole length, stem length, and shoot length. Furthermore, the shoot and stem lengths exhibited positive relationships with the petiole length, while showing negative relationships with the leaf area. Outstandingly, certain photosynthetic traits (levels of chlorophyll a, b, carotenoids, and greenness index) were positively associated with the root diameter growth rate.

The first two principal components (PC1 and PC2) accounted for 52.9% of the total data variability (Figure 7B). On the whole, ginseng plants treated with 20 PPFD are located in the right part of the PC1 scatter plot, and they possess higher root growth rates, stem diameters, bigger leaves, and greater photosynthetic traits. In contrast, 200 PPFD-treated samples are distributed in the left quadrants of the PC1 scatter plot. Notably, the ginseng plants grown in the 20/20 °C regime with 20 PPFD are mainly grouped in the upper right quadrant, presenting greater root and leaf growth, as well as photosynthesis. Consistent with the results above, 200 PPFD-cultivated plants displayed greater shoot, stem, and petiole lengths.

## 4. Discussion

Traditionally, the cultivation of ginseng requires a commitment to the long production growth cycle, and an optimal temperature combined with shade to thrive [4,32]. However, *Panax ginseng* sprouts (PGS) featuring short growth periods have been extensively cultivated as a marketable medicinal vegetable [5,6,33]. This study therefore adopted PGS as the experimental material and determined the DIF together with light intensity in a short-term cultivation (5 weeks).

The DIF treatments dramatically affected the plant height, as demonstrated by a great deal of pioneer research, such as that in cucumber, tomato, *Chrysanthemum*, etc. [12,34,35,36], whereas relevant studies on *Panax ginseng* sprouts remain scarce. In this trial, four DIF regimes, corresponding to positive, negative, and zero DIFs, were compared, which were readily accessible for the occurrences of distinct plant responses (Figure 1). Additionally, light intensity is one of the light components that greatly influence plant photosynthesis and affects agricultural production [37]. We subsequently applied 20 PPFD, as used in commercial ginseng production, while 200 PPFD was used for the recovery of ginseng seedling growth. The distinct responses of PGS to low and high light intensities were recorded due to the fact that the responses of plants to different light regimes (radiations and intensities) vary among species and are associated with culture conditions [37,38].

Given that many earlier studies on ginseng concentrated on the alleviation of abiotic stresses [39], disclosed certain regulating mechanisms [26,40,41], or only revealed one specific optimal culture parameter (temperature, light intensity, or quality) [6,22,28], the effects of simultaneous applications of DIF and LI on PGS are still not well known. Thus, the present study was designed and conducted to investigate the optimal culture conditions of DIF and LI for PGS. Consequently, in this study, zero DIF (20/20 °C) combined with 20 PPFD was shown to yield the best PGS growth.

### 4.1. Growth Parameters

PGS growth and morphology were considerably affected by the DIF and LI treatments (Figure 1). The root and shoot growth rate in response to the negative DIF (18/22 °C) was significantly slower than in response to the zero DIF and positive DIF, which is parallel to the former studies investigating tomato [42] and *Platycodon grandiflorum* [43]. This indicated that negative DIF (18/22 °C) hampered the growth of PGS, as reflected by the lowest root-, shoot-, and leaf-related diameters. Additionally, the root growth rate in fresh weight and leaf area were more pronounced in response to zero DIF (20/20 °C) than in response to positive DIF (22/18 °C) (Figure 2 and Figure 4). However, PGS exhibited better growth performance at 20/20 °C than at 22/22 °C, which can be explained by the fact that *P. ginseng* is a perennial shade plant that displays hypersensitivity, and even growth retardation, when subjected to increasing and/or high temperatures [23,44,45].

It is noteworthy that the light saturation point for ginseng is 250 PPFD [22,46,47]. A threshold value at 200 PPFD for ginseng growth is recommended according to Jung’s report [26], whereas 20 PPFD is used extensively for commercial large-scale production of PGS [27]. However, our study showed that PGS cultivated with 200 PPFD was more prone to spindliness (lower root fresh weight and root diameter, smaller leaf area and shoot diameter, but higher shoot length and petiole length) than those grown with 20 PPFD (Figure 2, Figure 3 and Figure 4; Table 1). This phenomenon agrees with the reports by Jang et al. [24]. In our study, the reductions in the root growth parameters and leaf area with the 200 PPFD treatments may be attributed to the absorptions of excessive light in the zero-DIF regimes, probably resulting in the photoinhibition of photosynthesis [26]. As noted by Proctor and Palmer, the optimal light intensity for ginseng cultivation is determined by various factors, in particular by the combinations of the plant responses and the associated specific environments [48]. Accordingly, this study shows that zero DIF at 20/20 °C in combination with 20 PPFD rendered the best PGS growth among the four treatments tested in terms of root-, shoot-, and leaf-associated properties.

### 4.2. Photosynthesis Characteristics

Photosynthesis is a complex biochemical and biophysical process that determines biomass output, and pertains to the synthesis of photosynthetic pigments [38]. A positive relationship between photosynthetic ability and the chlorophyll contents has been observed and confirmed by many scholars [22,29,49,50]. The concentrations of photosynthetic pigments—chlorophyll a, chlorophyll b, and carotenoids—were individually investigated. Except for negative DIF, PGS treated with 20 PPFD had higher chlorophyll contents (Figure 6). Potentially, even though 200 PPFD is regarded as a safe factor that causes negligible photoinhibition and photobleaching, the synergistic effects of DIF on the pigments should also be considered, as mentioned above [22,46,47,48]. For instance, surprisingly, negative-DIF-grown plants showed higher photosynthetic pigment levels when treated with 200 PPFD than those treated with 20 PPFD (Figure 6), probably because the plant’s flexible adaptations to satisfy the photosynthetic demand by maximizing the capture of light were active in the 200 PPFD regime [24]. Alterations of light intensity lead to distinct changes in the contents of photosynthesis pigments, and the light intensity had a significant influence on the contents of chlorophyll a and chlorophyll b (Figure 6A,B).

We adopted a hand-held portable chlorophyll meter for the assessment of photosynthesis-related parameters, by means of which the relationships between the leaf greenness index and the contents of the chlorophylls could accordingly be deduced [51]. Consistent with the results of the chlorophyll contents, the ginseng plants had higher greenness indices when cultivated with 20 PPFD, with both the DIF and LI significantly impacting the leaf greenness index (Figure 5).

In summary, zero- and positive-DIF-cultivated PGS displayed greater contents of photosynthesis-related pigments, as well as greater greenness indices, suggesting that these conditions promote photosynthetic capacity.

## 5. Conclusions

In summary, negative DIF distinctly constricted growth- and photosynthesis-related characteristics. When grown in the zero DIF regime, the ginseng plants treated with 20 PPFD showed better performances in roots, shoots, leaves, and photosynthesis compared to those with 200 PPFD. In addition, plants in the zero DIF regime formed bigger leaves than their positive DIF counterparts. Additionally, 200 PPFD conferred the plants with spindliness, and they were characterized by a longer shoot length and stem length, while forming smaller roots and leaves, and displaying poor growth rates, and slim shoots and stems.

In addition, the correlation analyses revealed that the root growth rate (consisting of fresh weight and diameter) was positively correlated with the stem diameter, leaf traits (length, width, and area), and photosynthetic ability (levels of chlorophyll a, b, carotenoids, and greenness index), whereas it was negatively correlated with the petiole length, stem length, and shoot length. Therefore, the simultaneous application of zero DIF at 20/20 °C and 20 PPFD could be regarded as the optimal condition for the best growth of PGS, as observed in this study. However, further investigations are necessary in order to obtain more data regarding the biochemical components in PGS, such as total the saponin and ginsenoside levels.

## Figures and Tables

**Table 1:** Shoot-, leaf-, and root-related parameters as affected by the DIF and light intensity (LI).

DIF (A)	Light Intensity (B)	Shoot Fresh Weight (g)	Shoot Dry Weight (g)	Shoot Diameter (mm)	Leaf Length (cm)	Leaf Width (cm)	Root Dry Weight (g)
18/22 °C	20	0.8 ± 0.16 a ^z^	0.08 ± 0.01 b	1.7 ± 0.11 a	4.2 ± 0.26 b	2.2 ± 0.21 a	0.19 ± 0.02 a
	200	0.9 ± 0.21 a	0.13 ± 0.02 a	1.8 ± 0.15 a	4.6 ± 0.28 a	1.9 ± 0.20 b	0.12 ± 0.01 b
20/20 °C	20	1.0 ± 0.13 a	0.14 ± 0.02 a	1.9 ± 0.11 a	4.8 ± 0.16 b	2.6 ± 0.18 a	0.23 ± 0.02 a
	200	0.8 ± 0.20 ab	0.12 ± 0.01 ab	1.7 ± 0.13 ab	5.0 ± 0.17 a	2.0 ± 0.17 b	0.18 ± 0.01 b
22/22 °C	20	0.9 ± 0.11 a	0.12 ± 0.01 a	1.8 ± 0.09 a	4.7 ± 0.21 b	2.4 ± 0.17 a	0.20 ± 0.02 a
	200	0.8 ± 0.13 a	0.09 ± 0.01 b	1.6 ± 0.10 ab	5.0 ± 0.22 a	2.1 ± 0.17 b	0.17 ± 0.03 ab
22/18 °C	20	0.9 ± 0.20 a	0.10 ± 0.01 a	1.8 ± 0.14 a	4.1 ± 0.19 b	1.9 ± 0.16 b	0.21 ± 0.01 a
	200	0.8 ± 0.18 a	0.08 ± 0.02 b	1.7 ± 0.14 a	5.0 ± 0.21 a	2.3 ± 0.17 a	0.19 ± 0.01 a
*F*-test	A	ns ^y^	**	ns	ns	ns	***
	B	ns	*	*	*	*	ns
	A × B	ns	**	ns	*	*	*

^z^ Data are the means ± SD (*n* = 6 replicates) accompanied by different lowercase letters indicating significant differences at *p* ≤ 0.05; ^y^ The *F*-test values determined by two-way ANOVA of different treatments refer to ns *p* > 0.05, where * *p* < 0.05, ** *p* < 0.01, and *** *p* < 0.001.

## Data Availability

Data sharing is not applicable to this article.
